# Supplementary material for: Meiogyne oligocarpa (Annonaceae), a new species from Yunnan, China
Source: PeerJ. 2021 Apr 14;9:e10999. doi: 10.7717/peerj.10999 (PMC8054756; doi:10.7717/peerj.10999)
Supplement: Supplemental Information 2 — The data are given using the following format: taxon name, origin, collector(s) and collector number (herbarium acronym), matK, ndhF, ndhF-rpl32, rbcL, rpl32-trnL, trnL-F, ycf1 GenBank numbers. Missing data are noted with an —; newly generated sequences for this study are annotated with an*. [file peerj-09-10999-s002.docx]

Appendix 1. Voucher information and GenBank accession numbers for the 73 Annonaceae samples used in this study. The data are given using the following format: taxon name, origin, collector(s) and collector number (herbarium acronym), *matK*, *ndhF*, *ndhF-rpl32*, *rbcL*, *rpl32-trnL*, *trnL-F*, *ycf1* GenBank numbers. Missing data are noted with an —; newly generated sequences for this study are annotated with an*.

***Alphonsea boniana*** Finet & Gagnep., Chanthaburi Province, Thailand, *P. Chalermglin 540107* (HKU), AY518809, JQ723785, JQ723816, AY318965, —, AY319077, —; ***Ambavia gerrardii*** (Baill.) Le Thomas, *H. Sauquet 23* (P), AY220435, AY218168, —, —, —, AY220411 (intron) AY220358 (spacer), —; ***Anaxagorea silvatica*** R. E. Fr., Brazil, *P. J. M. Maas 8836* (U), AY743477, EF179280, —, AY743439, —, AY743458, —; ***Annickia chlorantha*** (Oliv.) Setten & Maas, Gabon, *M. S. M.* *Sosef* *1877* (WAG), AY841393, AY841401, —, AY841594, —, AY841671, —; ***Annona glabra*** L., cult., Utrecht University Botanic Garden (origin: Florida), *L. W.* *Chatrou 467* (U), DQ125050, EF179281, —, AY841596, —, AY841673, —; ***Cananga odorata*** (Lam.) Hook. f. & Thomson, Costa Rica, *L. W. Chatrou 93* (U), AY841394, AY841403, —, AY841602, —, AY841680, —; ***Dasymaschalon macrocalyx*** Finet & Gagnep., Thailand, *P. J. A. Keßler 3199* (L), EF179277, EF179290, —, AY841610, —, AY841688, —; ***Desmopsis schippii*** Standl., Costa Rica, Alajuela, *Alfaro 4572* (U), AY518805, JQ723786, —, AY319060, —, AY319174, —; ***Disepalum*** ***platypetalum*** Merr., Sumatra, *W. Takeuchi* *18201* (L), DQ125057, EF179292, —, AY841612, —, AY841690, —; ***Fenerivia chapelieri*** (Baill.) R. M. K. Saunders, Madagascar, Toamasina, *Ludovic & Rallotoarivony 221* (P), JF810375, JQ723788, —, JF810387, —, JF810399, —; ***Goniothalamus griffithii*** Hook. f. & Thomson, *P. J. A. Keßler 3188* (L), AY743484, EF179296, —, AY743446, —, AY743465, —; ***Greenwayodendron oliveri*** (Engl.) Verdc., Ghana, *C. C. H. Jongkind et al. 1795* (WAG), AY743489, AY841408, —, AY743451, —, AY743470, —; ***Haplostichanthus johnsonii*** F. Muell., Queensland, Australia, *B. P. M. Hyland 7142* (L), JQ723767, JQ723791, JQ723821, JQ723854, —, JQ723907, JQ723928; ***Hubera cerasoides*** (Roxb.) Chaowasku [= *Polyalthia cerasoides* (Roxb.) Benth. & Hook. f. ex Beddome], Saraburi Province, Thailand, *B. Xue & P. Chalermglin XB11* (HKU), AY518854, JQ723810, JQ723843, AY319017, JQ723896, AY319131, JQ723950; ***Maasia sumatrana*** (Miq.) Mols, P. J. A. Kessler & S. H. Rogstad, Sumatra, *SAN 143918* (SAN), AY518873, AY841418, —, AY319039, —, AY319153, —; ***Malmea dielsiana*** R. E. Fr., Peru, *L. W. Chatrou 122* (U), AY238964, AY841410, —, AY238955, —, AY319177, —; ***Marsypopetalum crassum*** (R. Parker) B. Xue & R. M. K. Saunders, Chachoengsao Province, Thailand, *P. Chalermglin 521212-1* (HKU), HQ286571, JQ723792, JQ723822, HQ286577, JQ723875, HQ286583, JQ723929; ***Meiogyne amicorum*** (A. C. Sm.) B. Xue & R. M. K. Saunders [= *Polyalthia amicorum* A. C. Sm.], Fiji, *T. G. Yuncker 16228* (NY), KF301021, KF611912, —, —, —, KF573503, —; ***Meiogyne amygdalina*** (A. Gray) B. Xue & R. M. K. Saunders [= *Polyalthia amygdalina* (A. Gray) Gillespie], Fiji, *A. C. Smith 5613* (A), KF301022, KF611913, —, —, —, KF573497, —; ***Meiogyne baillonii*** (Guillaumin) Heusden, New Caledonia, *H. S. MacKee 196* (L), JQ723768, JQ723793, JQ723823, JQ723855, JQ723876, JQ723908, JQ723930; ***Meiogyne bidwillii*** (Benth.) D. C. Thomas, Chaowasku & R. M. K. Saunders 1 [= *Fitzalania bidwillii* (Benth.) Jessup, P. J. A. Kessler & Mols], Queensland, Australia, *G. Sankowsky 4139* (BRI), JQ723764, JQ723789, JQ723818, JQ723851, JQ723872, JQ723904, JQ723925; ***Meiogyne bidwillii*** (Benth.) D. C. Thomas, Chaowasku & R. M. K. Saunders 2 [= *Fitzalania bidwillii* (Benth.) Jessup, P. J. A. Kessler & Mols], Queensland, Australia, *G. Sankowsky 3179* (BRI), JQ723765, —, JQ723819, JQ723852, JQ723873, JQ723905, JQ723926; ***Meiogyne cylindrocarpa*** (Burck) Heusden, Guam, *L. Raulerson & M. Mesngon 18331* (L), KF301023, KF611914, —, KF301029, —, KF573500, —; ***Meiogyne cylindrocarpa*** (Burck) Heusden subsp. ***cylindrocarpa*** 1, Sabah, Borneo, *C. E. Ridsdale DV­M1­1930* (L), AY518796, JQ723794, JQ723824, AY318981, JQ723877, AY319093, JQ723931; ***Meiogyne cylindrocarpa*** (Burck) Heusden subsp. ***cylindrocarpa*** 2, Queensland, Australia, *G. Sankowsky 3175* (BRI), JQ723769, JQ723795, JQ723825, JQ723856, JQ723878, JQ723909, JQ723932; ***Meiogyne dumetosa*** (Vieill. ex Guillaumin) Heusden, New Caledonia, *H. S. MacKee 38941* (L), KF301024, KF611915, —, KF301030, —, KF573501, —; ***Meiogyne*** ***gardneri***, Thailand, *S. Gardner et al. ST 2014* (L), KF301027, KF611918, —, KF301031, —, KF573504, KF301032; ***Meiogyne glabra*** Heusden, New Britain, *Barker & Vinas 66735* (L), JQ723772, —, JQ723828, JQ723859, JQ723881, JQ723912, JQ723935; ***Meiogyne habrotricha*** (A. C. Sm.) B. Xue & R. M. K. Saunders [= *Polyalthia habrotricha* A. C. Sm.], Fiji, *A. C. Smith 5614* (A), KF301025, KF611916, —, —, —, KF573498, —; ***Meiogyne hainanensis*** (Merr.) Bân, Hainan, China, *F. C.* *How 70628* (P), JQ723773, —, JQ723829, JQ723860, JQ723882, JQ723913, JQ723936; ***Meiogyne hainanensis*** (Merr.) Bân, cult. South China Botanical Garden, China, B. Xue XB293 (IBSC), MW024839*, MW024841*, MW024844*, MW024847*, MW024856*, MW0248450*, MW024853*; ***Meiogyne heteropetala*** (F. Muell.) D. C. Thomas, Chaowasku & R. M. K. Saunders [= *Fitzalania heteropetala* (F. Muell.) F. Muell.], Queensland, Australia, *G. Sankowsky 4140* (BRI)*,* JQ723766, JQ723790, JQ723820, JQ723853, JQ723874, JQ723906, JQ723927; ***Meiogyne hirsuta*** (Jessup) Jessup, Queensland, Australia, *G. Sankowsky 3151* (BRI), JQ723774, JQ723798, JQ723830, JQ723861, JQ723883, JQ723914, JQ723937; ***Meiogyne insularis*** (A. C. Sm.) D. C. Thomas, B. Xue & R. M. K. Saunders [= *Meiogyne stenopetala* subsp. *insularis* (A. C. Sm.) Heusden], Fiji, *O.* *Degener 14986* (L), KF301028, KF611919, —, —, —, KF573502, —; ***Meiogyne kanthanensis*** Ummul-Nazrah & J.P.C. Tan, Perak, Malaysia, Tan J.P.C. et al. FRI81800 (KEP), MW024837*, —, MW024842*, MW024845*, MW024854*, MW024848*, MW024851*; ***Meiogyne laddiana*** (A. C. Sm.) B. Xue & R. M. K. Saunders [= *Polyalthia laddiana* A. C. Sm.], Fiji, *J. Franklin 66* (SDSU), KF301026, KF611917, —, —, —, KF573499, —; ***Meiogyne lecardii*** (Guillaumin) Heusden, New Caledonia, *H. S. McKee 16292* (L), JQ723775, JQ723799, JQ723831, JQ723862, JQ723884, JQ723915, JQ723938; ***Meiogyne*** ***maxiflora***, Kanchanaburi, Thailand, *P. J. A Keßler 3219* (L), AY518797, JQ723802, JQ723835, AY319002, JQ723888, AY319115, JQ723942; ***Meiogyne mindorensis*** (Merr.) Heusden, Philippines, *A. C.**Podzorski SMHI76* (L), JQ723776, JQ723800, JQ723832, JQ723863, JQ723885, JQ723916, JQ723939; ***Meiogyne monosperma*** (Hook. f. & Thomson) Heusden, Malaysia, Negeri Sembilan, *S. H. Rogstad 920* (L), JQ723777, —, JQ723833, JQ723864, JQ723886, JQ723917, JQ723940; ***Meiogyne oligocarpa*** *B. Xue & Y.H. Tan*, Yunnan Province, China, Y.Y. Shao SYY26 (IBSC), MW024838*, MW024840*, MW024843*, MW024846*, MW024855*, MW024849*, MW024852*; ***Meiogyne pannosa*** (Dalzell) J. Sinclair, Kerala, India, *Indu 2457* (L), JQ723778, JQ723801, JQ723834, JQ723865, JQ723887, JQ723918, JQ723941; ***Meiogyne* *stenopetala*** (F. Muell.) Heusden subsp. *stenopetala*, Queensland, Australia, *G. Sankowsky 3193* (BRI), JQ723779, JQ723803, JQ723836, JQ723866, JQ723889, JQ723919, JQ723943; ***Meiogyne trichocarpa*** (Jessup) D. C. Thomas & R. M. K. Saunders 1 [= *Meiogyne cylindrocarpa* (Burck) Heusden subsp. *trichocarpa* Jessup], Queensland, Australia, *G. Sankowsky 3190* (BRI), JQ723770, JQ723796, JQ723826, JQ723857, JQ723879, JQ723910, JQ723933; ***Meiogyne trichocarpa*** (Jessup) D. C. Thomas & R. M. K. Saunders 2 [= *Meiogyne cylindrocarpa* (Burck) Heusden subsp. *trichocarpa* Jessup], Queensland, Australia, *G. Sankowsky 4141* (BRI), JQ723771, JQ723797, JQ723827, JQ723858, JQ723880, JQ723911, JQ723934; ***Meiogyne verrucosa*** Jessup, Queensland, Australia, *G. Sankowsky 3188* (BRI), JQ723780, JQ723804, JQ723837, JQ723867, JQ723890, JQ723920, JQ723944; ***Meiogyne virgata*** (Blume) Miq., Kalimantan, Borneo, *P. J. A. Keßler 2751* (L), AY518798, JQ723805, JQ723838, AY318982, JQ723891, AY319094, JQ723945; ***Miliusa indica*** Lesch. ex A. DC., cult., Royal Botanic Gardens, Sri Lanka, Peradeniya, *A. M. A. S. Attanayake QST646* (HKU), JQ723781, JQ723806, JQ723839, JQ723868, JQ723892, JQ723921, JQ723946; ***Mitrephora alba*** Ridl., cult., private garden (origin: Thailand), *P. Chalermglin 530706* (HKU), AY518855, JQ723807, JQ723840, AY318994, JQ723893, AY319106, JQ723947; ***Mkilua fragrans*** Verdc., cult., Utrecht University Botanic Garden (origin: Kenya), *L. W. Chatrou 474* (U), DQ125060, EF179303, —, AY841634, —, AY841712, —; ***Monocarpia euneura*** Miq., Indonesia, *J. W. F. Slik 2002–2931* (L), AY518865, AY841412, —, AY318998, —, AY319111, —; ***Monoon fuscum*** (King) B. Xue & R. M. K. Saunders [=*Enicosanthum fuscum* (King) Airy Shaw], Phetchaburi Province, Thailand, *B. Xue & P. Chalermglin XB6* (HKU), AY518787, JQ723787, JQ723817, AY318973, —, AY319085, —; ***Monoon lateriflorum*** (Blume) Miq. [= *Polyalthia lateriflora* (Blume) King], cult., Kebun Raya, Bogor, *A. M. A. S. Attanayake XX.D49a* (HKU), JQ723783, JQ723811, JQ723844, JQ723870, JQ723897, JQ723923, JQ723951; ***Mosannona costaricensis*** (R. E. Fr.) Chatrou, Costa Rica, *L. W. Chatrou 90* (U), AY743503, AY841413, —, AY743510, —, AY743496, —; ***Neo-uvaria telopea*** Chaowasku, Thailand, *T. Chaowasku 77* (L), JX544751, JX544778, —, JX544755, —, JX544783, JX544766; ***Onychopetalum periquino*** (Rusby) D. M. Johnson & N. A. Murray, Bolivia, *L. W.* *Chatrou 425* (U), AY518876, AY841414, —, AY319065, —, AY319179, —; ***Orophea*** sp., Kanchanaburi Province, Thailand, *P. Chalermglin 530207-1* (HKU), JQ723782, JQ723808, JQ723841, JQ723869, JQ723894, JQ723922, JQ723948; ***Phaeanthus splendens*** Miq., Borneo, *P. J. A. Keßler B 1564* (L), AY518864, JX544777, —, JX544754, —, AY319126, JX544765; ***Piptostigma mortehani*** De Wild., Gabon, *J. J.* *Wieringa 2779* (WAG), AY743492, AY841415, —, AY743454, —, AY743473, —; ***Platymitra macrocarpa*** Boerl., cult., Kebun Raya, Bogor, *Ardi WI 52* (HKU), AY518812, JQ723809, JQ723842, AY319013, JQ723895, AY319127, JQ723949; ***Polyalthia suberosa*** (Roxb.) Thwaites, Saraburi Province, Thailand, *B. Xue & P. Chalermglin XB12* (HKU), AY220439, AY841417, JQ723845, AF193971, JQ723898, AY319152, JQ723952; ***Popowia pisocarpa*** (Blume) Endl., Hong Kong, China, *B. Xue XB13* (HKU), AY518862, JQ723812, JQ723846, AY319044, JQ723899, AY319158, JQ723953; ***Pseudoxandra lucida*** R. E. Fr., *L. W. Chatrou et al. 212* (U), AY518870, AY841420, —, AY319076, —, AY319190, —; ***Pseuduvaria fragrans*** Y. C. F. Su, Chaowasku & R. M. K. Saunders, Surat Thani Province, Thailand, *T. Chaowasku 27* (HKU), JQ723784, JQ723813, JQ723847, JQ723871, JQ723900, JQ723924, JQ723954; ***Sageraea lanceolata*** Miq., Borneo, *C. E. Ridsdale DV-M2-1692* (L), AY518799, JX544774, —, AY319050, —, AY319164, JX544762; ***Sapranthus viridiflorus*** G. E. Schatz, Costa Rica, *P. J. M. Maas 7956* (U), AY743493, AY841422, JQ723848, AY319051, JQ723901, AY319165, JQ723955; ***Stelechocarpus burahol*** (Blume) Hook. f. & Thomson, cult., Singapore Botanic Garden, *N. Abdul Karim s.n.* (HKU), AY518803, JX544775, —, AY319053, —, AY319167, JX544763; ***Stenanona costaricensis*** R. E. Fr., Costa Rica, *L. W. Chatrou et al. 67* (U), AY518801, JX544772, —, AY319069, —, AY319183, JX544759; ***Tridimeris*** sp., cult., Missouri Botanical Garden, *P. J. M. Maas 8646* (U), JX544750, JX544773, —, JX544753, —, JX544782, JX544761; ***Trivalvaria costata*** (Hook. f. & Thomson) I. M. Turner [= *Trivalvaria dubia* (Kurz) J. Sinclair], cult., South China Botanic Garden, *M.Y. Wong 0805* (HKU), HQ286574, JQ723815, JQ723850, HQ286580, JQ723903, HQ286586, JQ723957; ***Wuodendron praecox*** (Hook.f. & Thomson) B. Xue, Y.H. Tan & X.L. Hou, Thailand, *T. Chaowasku 108* (L), JX544749, JX544770, —, JX544752, —, JX544781, JX544757; ***Uvaria lucida*** Benth., cult., Utrecht University Botanic Garden (origin: West Africa), *UUBG 84GR00334*, AY238966, EF179310, —, AY238957, —, EF179319, —; ***Xylopia peruviana*** R. E. Fr., cult., Utrecht University Botanic Garden, *L. W. Chatrou 483* (L), AY238967, EF179312, —, AY238958, —, AY231291, —.
